# Binding mode of Isoxazolyl Penicillins to a Class-A *β*-lactamase at ambient conditions

**DOI:** 10.1038/s42004-025-01801-x

**Published:** 2025-12-01

**Authors:** Gargi Gore, Andreas Prester, David von Stetten, Kim Bartels, Eike C. Schulz

**Affiliations:** 1 University Medical Center Hamburg-Eppendorf (UKE), Hamburg, Germany; 2 Institute for Nanostructure and Solid State Physics, University of Hamburg, Hamburg, Germany; 3 European Molecular Biology Laboratory (EMBL), Hamburg, Germany; 4 Max-Planck-Institute for the Structure and Dynamics of Matter, Hamburg, Germany

**Keywords:** X-ray crystallography, Drug discovery

## Abstract

The predominant resistance mechanism observed in Gram-negative bacteria involves the production of *β*-lactamases, which catalyse the hydrolysis of *β*-lactam antibiotics, thereby rendering them ineffective. Although Isoxazolyl Penicillins have been available since the 1970s, there are currently no structures in complex with class-A *β*-lactamases available. Here we have analysed the structure of the clinically relevant *β*-lactamase CTX-M-14 from *Klebsiella pneumoniae* near physiological temperatures, via serial synchrotron crystallography. We demonstrate the acyl-enzyme intermediates of the catalytically impaired CTX-M-14 mutant E166A in complex with three Isoxazolyl-Penicillins: Oxacillin, Cloxacillin and Dicloxacillin. Structural comparisons of CTX-M-Penicillin complexes show that, while conserved active-site interactions are maintained, each Isoxazolyl-Penicillin adopts a distinct conformation. While the three derivatives differ only by one and two chlorine atoms, respectively, their conformational heterogeneity appears to be increased by chlorination of the phenyl ring.

## Abstract (web summary)

Class-A *β*-lactamases can render antibiotics ineffective through hydrolysis, but the associated enzyme-antibiotic complexes remain largely underexplored at room temperatures. Here, the authors use RT serial synchrotron crystallography to report acyl-enzyme intermediates of the catalytically impaired *β*-lactamase CTX-M-14 mutant (E166A) from Klebsiella pneumoniae, and show that different isoxazolyl-penicillins adopt different conformations at room temperature.

## Introduction

Bacterial resistance to antibiotics is a leading cause of millions of deaths every year. About 13.6% of the total annual global infection-related deaths are associated with 33 bacteria^1^. Since the discovery of penicillin almost a century ago, antibiotics, especially *β*-lactams, remain irreplaceable. *β*-lactams are the most important as well as the most widely prescribed class of antibiotics against Gram-negative infectious bacteria^2^.

Most bacteria have an innate resistance to certain antibiotic groups as a result of low affinity, cell wall impermeability, and absence of antibiotic receptor^3^. Changes to this susceptibility can either be caused by spontaneous chromosomal mutations leading to the acquisition of resistance genes or the development of an extrachromosomal resistance mechanism. As a result of a Horizontal Gene Transfer (HGT), acquired extrachromosomal antibiotic resistance is usually contained within mobile DNA elements of the bacteria. Hence, mobile genetic elements like plasmids, conjugative transposons, and integrons are a driving force for bacterial multidrug resistance^4,5^. Among the numerous bacterial resistance mechanisms, the most prevalent include enzymatic modification of the antibiotic, target site modification, generation of an alternative metabolic pathway, increasing concentration of an antagonist of the antibiotic, or active removal of the antibiotic from the cell^3^.

The primary mode of resistance in Gram-negative bacteria is enzymatic degradation of antibiotics by *β*-lactamases^2,6^. The bacterial cell wall comprises heavily cross-linked peptidoglycans. These individual peptidoglycans are produced in the cell, but the cross-linking takes place beyond the cytoplasmic membrane, catalysed by cell-wall transpeptidases. Transpeptidases have an active site serine and proceed via an acylation-deacylation pathway to link two peptide strands. *β*-lactam antibiotics effectively inhibit this catalytic cycle due to the stereochemical resemblance of the *β*-lactam with the terminal peptide residues involved in the cross-linking. Thus, the transpeptidases form an irreversible penicilloyl-enzyme complex, thereby blocking peptidoglycan cross-linking during cell wall synthesis, increasing the likelihood of cell lysis and death^2^. Therefore, these transpeptidases are also referred to as penicillin-binding proteins (PBPs)^7^.

**Fig. 1:**
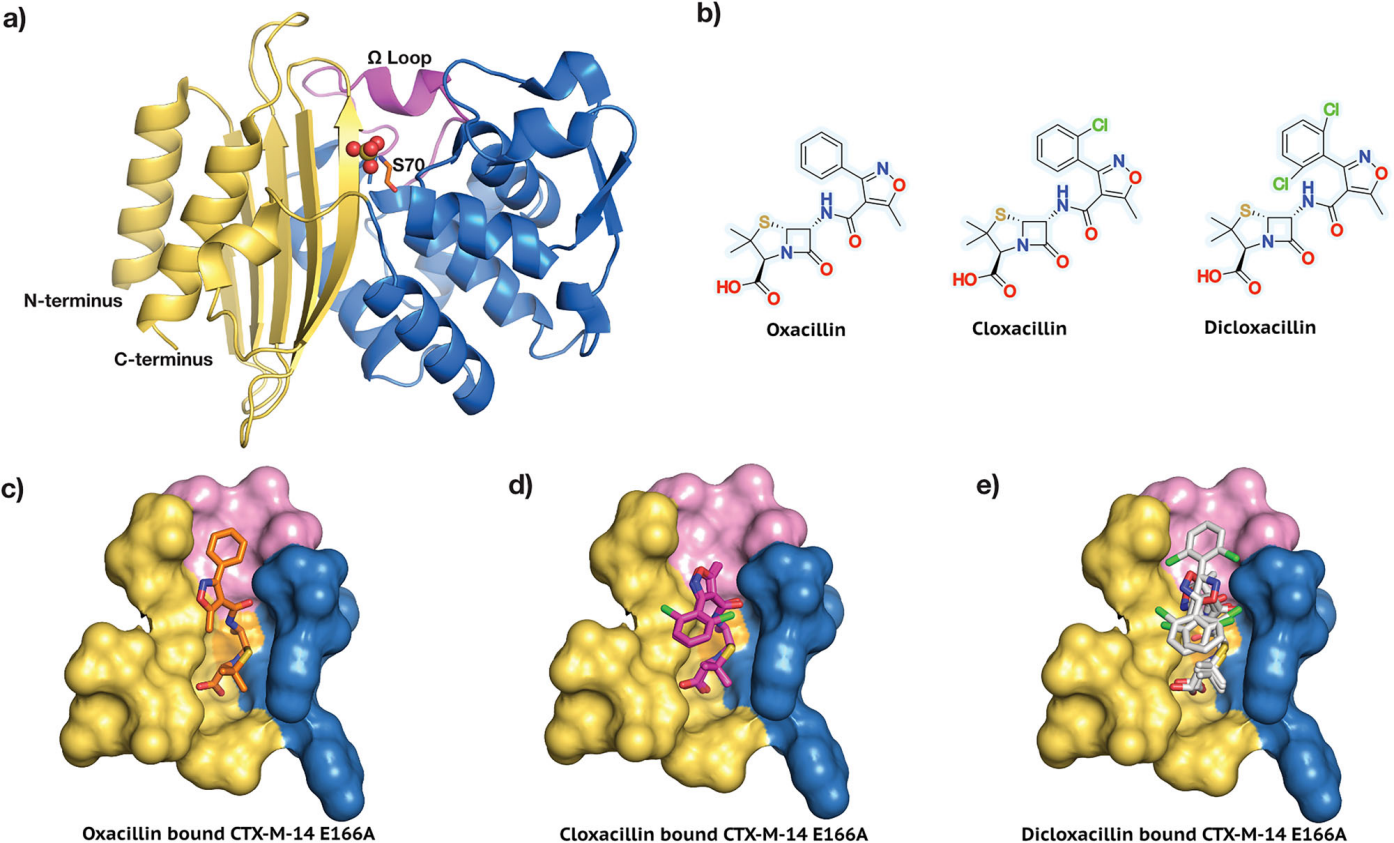
CTX-M-14 in complex with Isoxazolyl Penicillins. **a** CTX-M-14 *β*-lactamase. The catalytically important active site residue Ser 70 and the functionally relevant *Ω* loop (residues 163–176) are highlighted. **b** The Isoaxazolyl Penicillins, Oxacillin, Cloxacillin, and Dicloxacillin are shown in their unhydrolyzed form. **c** Oxacillin in the CTX-M-14 E166A active site. **d** Cloxacillin in the CTX-M-14 E166A active site. **e** Dicloxacillin in the CTX-M-14 E166A active site.

To avoid this, bacteria produce *β*-lactamases, enzymes that hydrolyse the *β*-lactam amide, rendering it ineffective in mimicking the cross-linking terminal peptides. The number of identified *β*-lactamases has greatly increased over the years; at the time of writing, the *β*-Lactamase Data-Base^8^ shows 8273 identified enzymes. Over the years, extensive use of broad-spectrum *β*-lactams, especially cephalosporins against Enterobacteriaceae since the 1980s, has led to selection pressure on bacteria, eventually giving rise to Extended Spectrum *β*-lactamases (ESBLs). Generally, ESBL-producing bacteria are inherently resistant to most penicillins, first-, second-, and third-generation cephalosporins as well as aztreonam. However, most ESBLs typically do not hydrolyse carbapenems, and can be inhibited by some *β*-lactamase inhibitors^4,9^. Enterobacteriaceae family members have adapted two methods for ESBL evolution: (i) selection of mutants having inherent wide substrate specificity (SHV- and TEM-type-*β*-lactamases) and (ii) acquisition of novel *β*-lactamase genes from the surrounding metagenome^10^. CTX-Ms (cefotaximase) have become the most successful ESBLs of the latter type. They belong to plasmid-encoded Ambler class A active-site serine ESBLs. The most common hosts for this acquired expression of CTX-M type enzyme have been *Escherichia coli* and *Klebsiella pneumoniae*, but it has also been reported in *Salmonella enterica, Shigella spp., Klebsiella oxytoca, Enterobacter spp., Pantoea agglomerans, Citrobacter spp., Serratia marcescens, Proteus mirabilis, Morganella morganii*, and *Providencia spp*.^10^. Out of all the CTX-M sub-lineages, CTX-M-1 and CTX-M-9 clusters are the most widespread. CTX-M-14 (Fig. 1a) belongs to the CTX-M-9 cluster and is one of the most prevalent members of the group^10^. CTX-M-14 *β*-lactamase hydrolyses most cephalosporins and penicillins as well as carbapenems, although the latter with lower efficiency^11^. Despite their long prevalence, structural information for Isoxazolyl antibiotics in complex with Class-A *β*-lactamases is still missing.

Isoxazolyl Penicillins are a class of semi-synthetic antibiotics with effective oral absorption, first produced and tested in the mid-1960s^12,13^. This class contains four major antibiotics: Oxacillin, Cloxacillin, Dicloxacillin, and Flucloxacillin.

They are synthesised by reacting an Isoxazolyl group with the 6-amino-penicillanic acid nucleus derived from penicillin G^14^. These are acid-stable and resistant to penicillinase enzymes produced by Gram-positive bacteria. For instance, the therapeutic efficacy of the Isoxazolyl Penicillins in treating staphylococcal and streptococcal infections was well established in the 1970s^15^. These narrow-spectrum antibiotics, especially Flucloxacillin and Oxacillin, are commonly prescribed against skin and soft tissue infections and osteomyelitis^16^. In an in-vitro synergism study against 40 strains of 9 *β*-lactamase producing Gram-negative Enterobacteriaceae members, including E. coli and *K. pneumoniae*, Oxacillin, Cloxacillin, and Dicloxacillin were paired with mezlocillin, a semi-synthetic ureidopenicillin. The combination was successful for strains of *Morg. morganii, E. coli, Ser. marcescens*, but showed no activity against *Klebsiella, Citrobacter* or *Enterobacter*^17^. Kinetic parameters of *β*-lactamases turning over Isoxazolyl Penicillins listed in the BRENDA-enzymes databases vary by several orders of magnitude for both *k*_*cat*_ and *K*_*M*_ values, for Oxacillin and Cloxacillin, depending on the *β*-lactamase class, mutation, and experimental conditions^18^. For Dicloxacillin, no kinetic parameters are listed in the database. While not specifically CTX-M-14, kinetic parameters for class A *β*-lactamases have been reported with *k*_*cat*_ of 105/s and *K*_*M*_ of 0.04 mM for Oxacillin and *k*_*cat*_ of 90/s and *K*_*M*_ of 0.04 mM for Cloxacillin^19^, respectively.

To understand the binding mode and the influence of the derivatisation in Isoxazolyl antibiotics, we aimed to gain structural insight into a stable enzyme-substrate complex. As there is currently no structure of a Class-A *β*-lactamase in complex with Isoxazolyl Penicillins available, we utilised the activity-impaired mutant CTX-M-14 E166A. This mutant traps the acyl-enzyme intermediate by covalently binding the Isoxazolyl Penicillins to the catalytic serine residue (Ser 70). Recent multi-temperature crystallography studies suggest that ambient or physiological temperatures permit resolving a larger conformational ensemble than under cryo-conditions^20–23^. In other words, data collection closer to physiological temperatures could aid in capturing lowly populated conformational states potentially relevant to ligand binding or catalysis. Making use of our recently developed serial crystallography environmental control box^24^, we successfully obtained serial room-temperature crystal structures of Oxacillin, Cloxacillin, and Dicloxacillin bound to CTX-M-14 E166A (Fig. 1c–e).

## Results and Discussion

### Shared Isoxazolyl-Penicillin active-site interactions with CTX-M-14

**Fig. 2:**
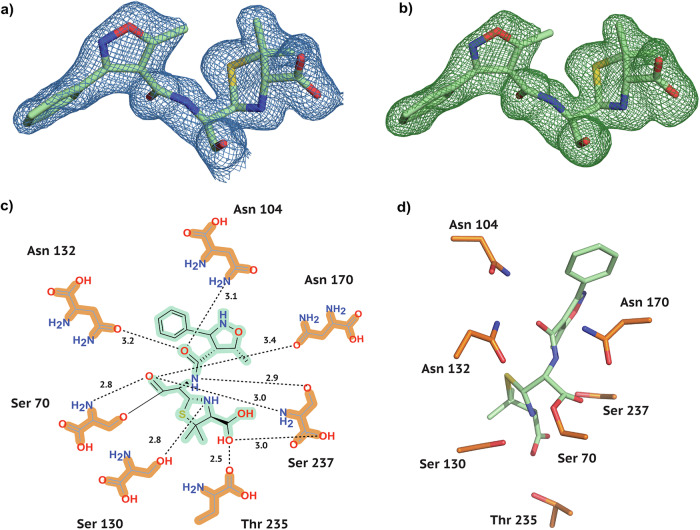
Electron density and conformation of Oxacillin at 20 °C. **a** 2F_*o*_-F_*c*_ map shown at an RMSD of 1.0. **b** POLDER omit map shown at an RMSD of 3.0. **c** 2D projection of Oxacillin and the contact residues at 20 °C showing interatomic distances. **d** Oxacillin conformation at 20 °C in the CTX-M-14 active site. Hydrogen bonds are represented as black dotted lines with their distance given in Å.

**Fig. 3:**
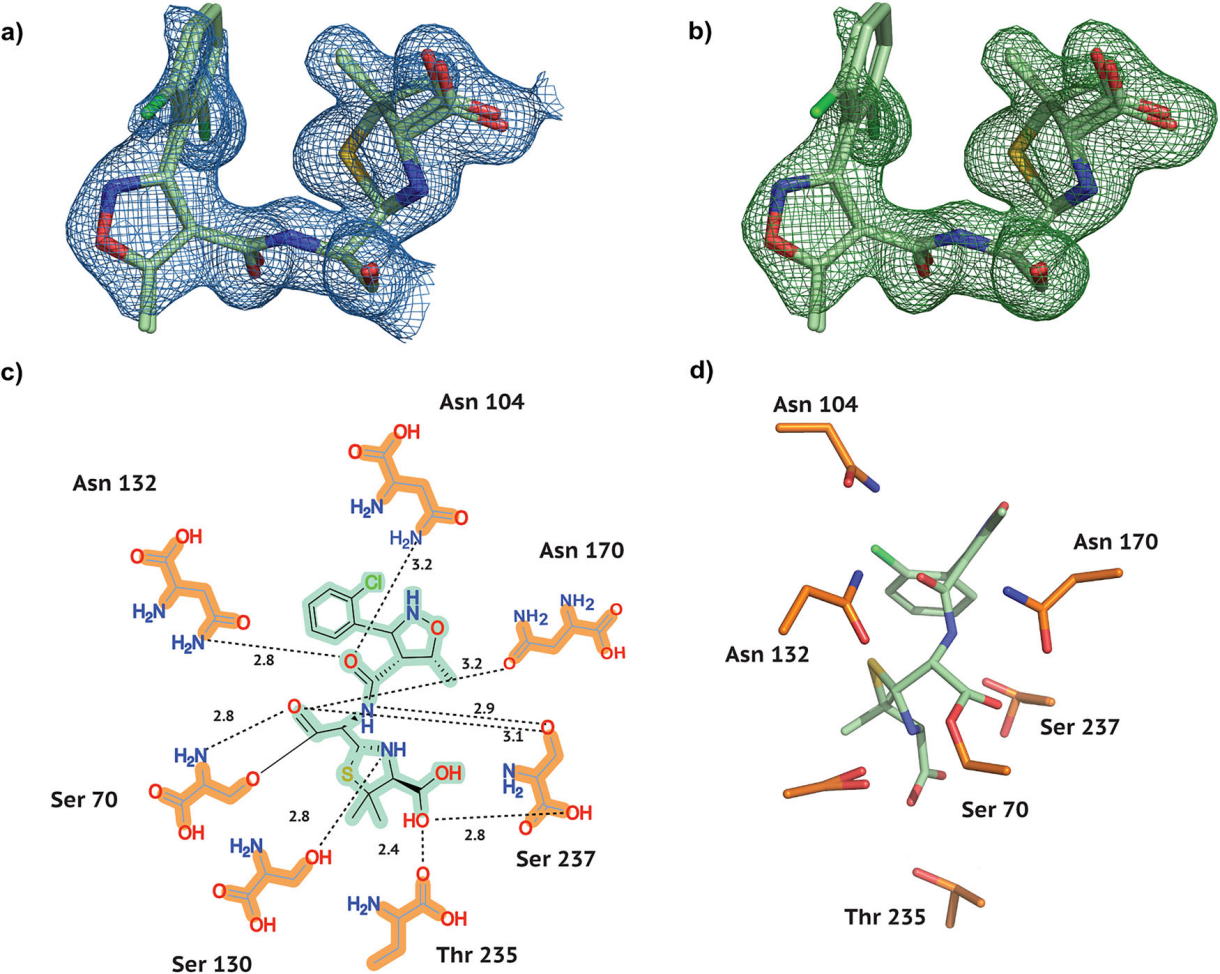
Electron density and conformation of Cloxacillin at 20 °C. **a** Two modelled conformations with a 2F_*o*_-F_*c*_ map shown at an RMSD of 1.0. **b** Two modelled conformations with POLDER omit map shown at an RMSD of 3.0. **c** 2D projection of Cloxacillin and the contact residues at 20 °C showing interatomic distances. **d** Cloxacillin conformation at 20 °C in the CTX-M-14 active site. Hydrogen bonds are represented as black dotted lines with their distance given in Å.

**Fig. 4:**
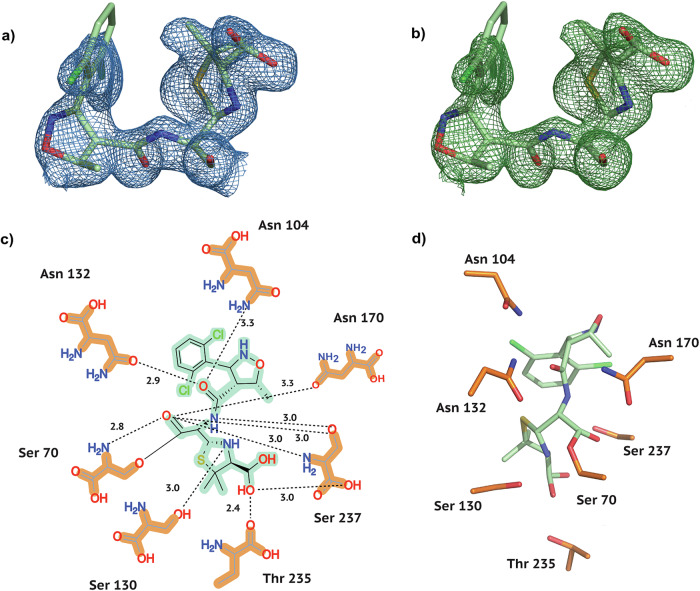
Electron density and conformation of Dicloxacillin at 20 °C. **a** 2F_*o*_-F_*c*_ map shown at an RMSD of 1.0. **b** POLDER omit map shown at an RMSD of 3.0. **c** 2D projection of Dicloxacillin and the contact residues at 20 °C showing interatomic distances. **d** Dicloxacillin conformation at 20 °C in the CTX-M-14 active site. Hydrogen bonds are represented as black dotted lines with their distance given in Å.

Using room temperature serial synchrotron crystallography (SSX), we have obtained the crystal structures of the Class A *β*-lactamase CTX-M-14 mutant E166A, bound to three Isoxazolyl Penicillins, namely Oxacillin, Cloxacillin, and Dicloxacillin (Figs. 2–4). All crystal structures were obtained at a comparable resolution of about 1.7 Å and could be refined to reasonable data-quality parameters (Table 1).

**Table 1:** Data collection and refinement statistics of the CTX-M-14 and Isoxazolyl complexes at room temperature

Ligand (PDB-ID)	Oxacillin (9R0R)	Cloxacillin (9RAO)	Dicloxacillin (9RAJ)
*Data collection*			
Space group	P3_2_21	P3_2_21	P3_2_21
Cell dimensions			
*a, b, c* (Å)	41.98, 41.95, 234.20	42.20, 42.20, 234.53	41.90, 41.87, 234.06
*α*, *β*, *γ* (^∘^)	89.99, 89.98, 120.02	89.99, 89.99, 119.93	89.99 89.98 120.03
Resolution range (Å)	117.65 - 1.70 (1.70–1.76)	117.65 - 1.70 (1.70–1.76)	117.65 - 1.70 (1.70–1.76)
Diffraction patterns	7405	4775	11,145
Total reflections	1,225,614 (71313)	1,944,328 (113101)	2,597,656 (175215)
Unique reflections	27,980 (2742)	27,982 (2731)	27,982 (2734)
Redundancy	43.8 (26.1)	139.0 (41.4)	92.8 (55.3)
Completeness (%)	100 (100)	100 (100)	100 (100)
Mean I/*σ* (I)	2.65 (0.66)	5.46 (0.86)	4.04 (1.74)
Wilson B-factor (Å^2^)	20.61	20.36	19.73
*R*_split_	0.323 (1.082)	0.219 (1.247)	0.163 (1.595)
CC1/2	0.8609 (0.409)	0.883 (0.257)	0.938 (0.535)
CC*	0.9619 (0.762)	0.969 (0.639)	0.984 (0.835)
*Refinement*			
Reflections used in refinement	23,606 (2424)	23,606 (2423)	23,611 (2427)
*R*_work_	0.2109(0.3008)	0.1794 (0.2785)	0.1605 (0.2221)
*R*_free_	0.2464 (0.3376)	0.1816 (0.3518)	0.1700 (0.2890)
Reflections used for *R*_free_	1229 (131)	1230 (131)	1229 (131)
*Number of non-hydrogen atoms*	2331	2396	2362
macromolecules	2139	2154	2156
ligands	28	58	29
solvent	164	193	181
*Average B-factor* (Å^2^)	36.58	29.28	24.00
macromolecules	35.32	28.21	23.20
ligands	49.55	30.60	23.21
solvent	43.45	40.71	34.97
*RMS deviations*			
Bond lengths (Å)	0.002	0.004	0.013
Bond angles (^∘^)	0.861	0.702	1.573

Values in the highest resolution shell are shown in parentheses.

As is generally characteristic of penicillin antibiotics, our three substrates contain a thiazolide ring attached to a 4-membered *β*-lactam ring (Fig. 1b). Furthermore, in Isoxazolyl Penicillins, the amide branch on the *β*-lactam ring opposite to the nitrogen has a variable sidechain, bearing a 3-phenyl-5-methylisoxazole in Oxacillin, a 3-(2-chlorophenyl)-5-methylisoxazole in Cloxacillin, and 3-(2,6-dichlorophenyl)-5-methylisoxazole in Dicloxacillin. After structure solution, successful complex formation with the ligands was clearly evident from the strong difference electron density connected to the catalytically active site residue Ser 70. In addition, typical ligand-residue interactions in the binding pocket of CTX-M-14 were also observed for all three ligands. Commonly, the ligand poses are stabilised by hydrogen bonds between the ligand and the surrounding Ser 70, Asn 104, Ser 130, Asn 132, Asn 170, Thr 235, and Ser 237 residues (Figs. 2–4).

In class A *β*-lactamases, the main chain nitrogens of residues Ser 70 and Ser 237 form hydrogen bonds with the carbonyl oxygen of the *β*-lactam; this is thought to stabilise the developing negative charge on the tetrahedral intermediate during the acylation step^25^. This is also observed in all complexes, where 2.8–2.9 Å and 3.0 Å hydrogen bonds are maintained between the *β*-lactam carbonyl and the main chain nitrogens of Ser 70 and Ser 237, respectively.

Previously reported key interactions within the *Ω* loop (Fig. 1a) of Class A ESBLs are also observed in all three CTX-M-14 E166A ligand complexes^26,27^, for instance, the H-bonds between Arg 164 and Thr 171, Ala 166 and Asn 136 (contrary to Glu 166 and Asn 170 in the native enzyme). Similarly, salt bridges between Arg 164 and Asp 179, Arg 161 and Asp 163, Asp 176 and Arg 178 are observed. These interactions are vital for maintaining the structural integrity of the *Ω* loop, which encloses the CTX-M active site. There are no major conformational differences in the *Ω* loop between the room temperature structures of our three Isoxazolyl-CTX-M-14 E166A complexes.

At the time of writing, ten structures of Oxacillin (Oxacillin derivatives are excluded) and two of Cloxacillin bound to *β*-lactamases and PBPs are deposited in the PDB. On the other hand, there are only two available structures of Dicloxacillin in the PDB, and neither is bound to a *β*-lactamase or PBP. Notably, there are no PDB entries of the Isoxazolyl Penicillins in complex with a class A *β*-lactamase. Furthermore, all deposited structures are collected at cryo-temperatures (Table 2).

**Table 2:** List of PDB entries with Oxacillin, Cloxacillin, or Dicloxacillin, respectively

Ligand	Class B	Class C	Class D	PBPs	Non-*β*-lactamases
Oxacillin	4EYB	4JXG^†^	4F94, 7O5Q^†*^,	8VUB, 5OJ1,	-
			7PSE, 4MLL	5OIZ, 5M19	-
Cloxacillin	-	1FCM	-	3MZD	-
Dicloxacillin	-	-	-	-	4R23, 6ZOA

^†^*lacking accompanying publication*.

*additional Oxacillin derivative present.

### A distinct Oxacillin binding mode in CTX-M-14

Plasmid-born Oxacillin resistance was first mentioned in the late 1960s, and it was hypothesised that this resistance originated from an Oxacillin-hydrolysing *β*-lactamase^28^. Consequently, these enzymes were referred to as Oxacillinases, due to their preferential substrate selection of isoxazolyl-type penicillins. Today, Oxacillinases belong to class D *β*-lactamases and have evolved to become a diverse family with broad substrate profiles including penicillins, extended-spectrum cephalosporins, and even carbapenems^2,29,30^.

To compare our room-temperature Oxacillin-bound CTX-M-14 E166A complex (Fig. 2) to previously solved Oxacillin complexes, we selected the class D *β*-lactamase OXA-1 structure (Table 2, PDB entry 4MLL)^30^. OXA-1 is an important subtype of Ambler Class D enzymes, which effectively hydrolyse Oxacillin. Its 3D structure was previously solved at a resolution of 1.4 Å, and it shares 16.5% sequence identity with CTX-M-14 E166A, while the other published class D enzymes in complex with Oxacillin, 7PSE^31^, and 4F94^30^ share only 6.4% and 13.5% sequence identity, respectively. Since the Class B New Delhi metallo-*β*-lactamase (PDB entry 4EYB)^32^ displays a different binding mode and lower homology to CTX-M-14 (13.2% sequence identity), this structure, as well as the Class C and Class D entries that lack an accompanying publication, were not considered for a detailed comparison.

OXA-1 (4MLL) has four monomers, out of which 3 show bound Oxacillin at the active site. Monomer C was chosen for comparison, having the lowest B factors as well as the least amount of unmodelled difference density features around the ligand in the deposited structure. In OXA-1, the carboxyl oxygen of the *β*-lactam ring forms H-bonds with the main-chain nitrogens of Ala 215 and active site Ser 67, which correspond to Ser 237 and active Ser 70 in CTX-M-14. Along the same lines, the amide nitrogen of the variable side-chain moiety forms an H-bond with the backbone carbonyl of Ala 215 in OXA-1, while in CTX-M-14 it is connected to Ser 237. Usually, the amide oxygen interacts with a polar residue (Asn 104 and Asn 132 in CTX-M-14) in *β*-lactamases. However, in OXA-1, a hydrogen bond with a water molecule is observed, due to the hydrophobic Val 117 in place of a polar residue. The carboxylate of the thiazolide ring forms hydrogen bonds with Ser 258, Thr 213, and waters in OXA-1, while the polar contacts to water molecules vary between the four OXA-1 monomers in the asymmetric unit. In CTX-M-14, on the other hand, Thr 235 and Ser 237 interact with the thiazolide ring. It has been reported that class D enzymes possess a more hydrophobic active site than class A enzymes^33^, which complements the largely hydrophobic moiety of Oxacillin^30^. Nevertheless, we have observed a stable Class A *β*-lactamase–Oxacillin complex with unambiguous electron density, further underlining the broad substrate spectrum of class A ESBLs.

**Fig. 5:**
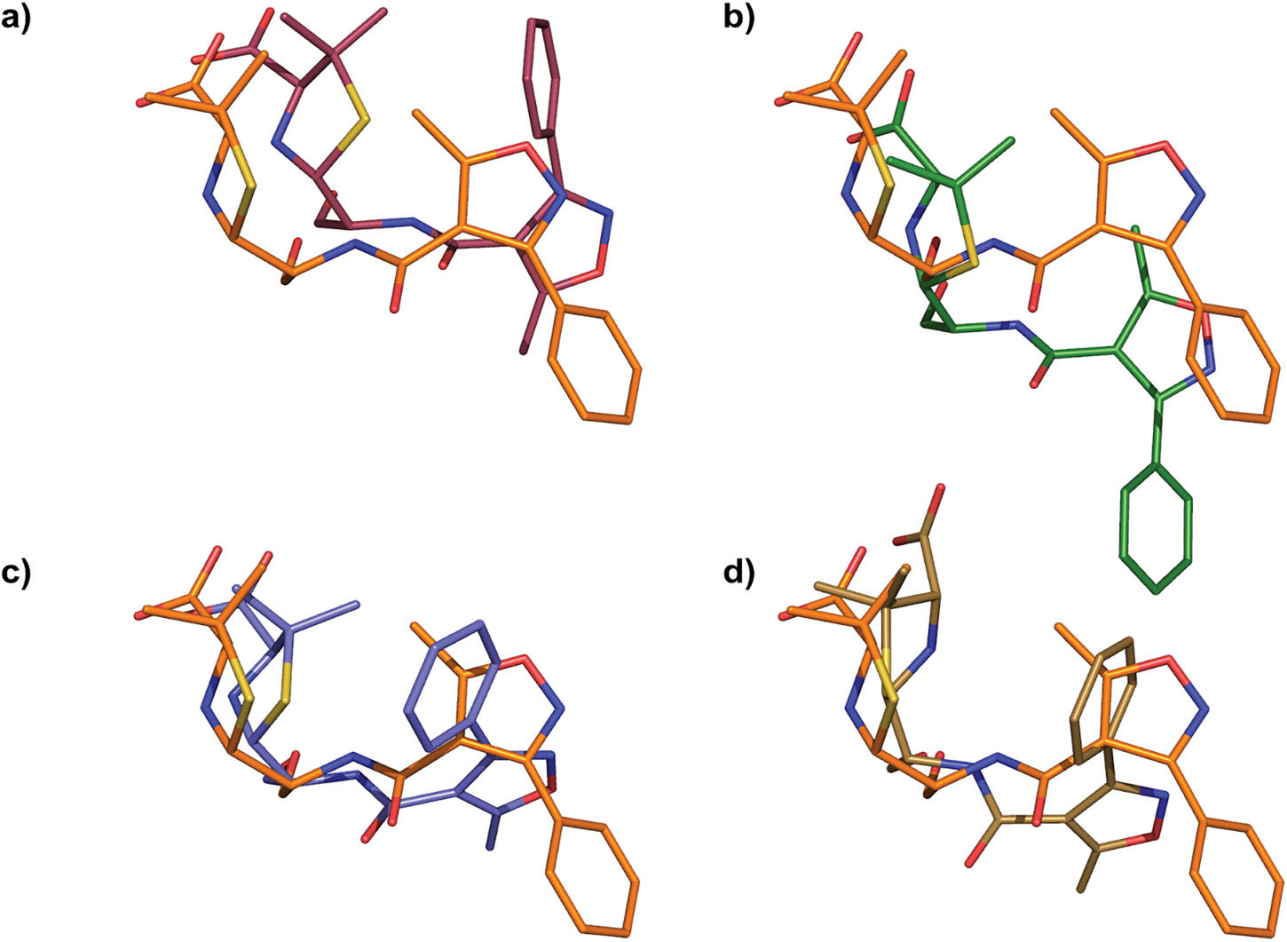
Comparison of Oxacillin orientations in the active site. **a**–**d** CTX-M-14 E166A bound Oxacillin, PDB entry 9R0R (orange) superposed to Oxacillin from PDB entry (**a**) 4JXG (Class C), (**b**) 4F94 (Class D), (**c**) 4MLL (Class D), (**d**) 7PSE (Class D).

To illustrate localised difference in the binding mode of Oxacillin with different *β*-lactamases, we superposed our CTX-M-14 E166A-bound Oxacillin with other class C and class D *β*-lactamase-bound Oxacillin molecules (Table 2) in the PDB (Fig. 5a–d). Interestingly, in contrast to all previously studied complexes, our room-temperature structure reveals a different binding pose for Oxacillin. While the 3-phenyl-5-methylisoxazole moiety is oriented toward Pro 167 of CTX-M-14 E166A (Fig. 5a–d), this group is rotated by 90–150° in other Oxacillin-bound structures. Whether this difference is rooted in the structural differences of Class A *β*-lactamases or can be attributed to the higher data-collection temperature will be explored in future studies.

### Two distinct Cloxacillin conformations in CTX-M-14

**Fig. 6:**
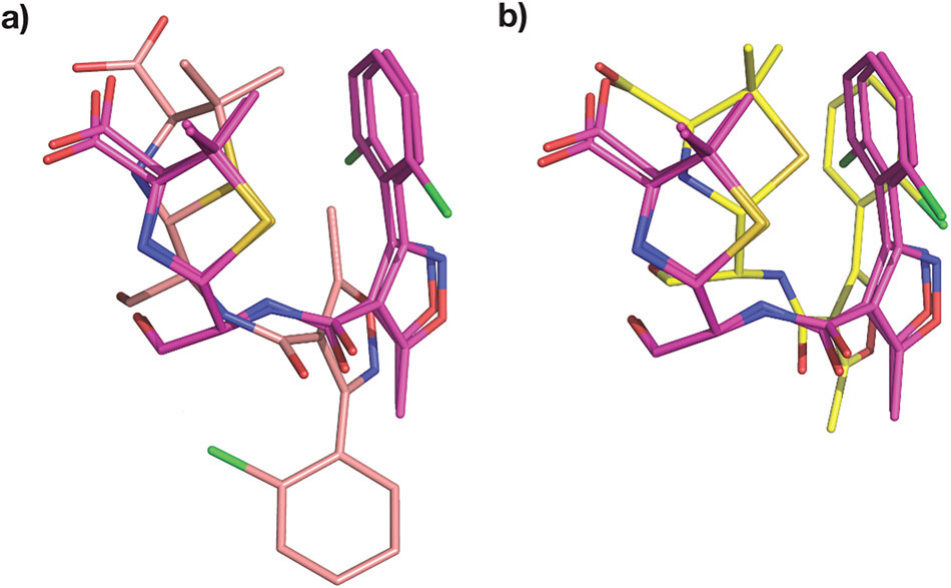
Comparison of Cloxacillin orientation in the active site. Figures (**a**, **b**) show CTX-M-14 E166A bound Cloxacillin, PDB entry 9RAO (magenta) superposed to Cloxacillin from PDB entry (**a**) 1FCM (Class C) (**b**) 3MZD (PBP-5).

To compare our Cloxacillin complex to previously solved structures, we selected the complex with Class C *β*-lactamase AmpC^34^ (PDB entry 1FCM, 2.46 Å resolution, 14.5% sequence identity) from E. coli, and the E. coli Penicillin-Binding Protein 5^35^ (PDB entry 3MZD, 1.90 Å resolution, 12.2% sequence identity). Cloxacillin-PBP-5 forms multiple H-bonds between the carboxyl of the thiazolide ring and residues Arg 248 and Thr 214. Additionally, the main chain nitrogens of the active Ser 44 and Gly 215 form H-bonds with the carboxyl oxygen of the *β*-lactam ring. The amide branch of the *β*-lactam ring, to which the variable moiety is attached, forms multiple H-bonds with Ser 87 and Asn 112. Along similar lines, in the Cloxacillin-AmpC structure, the thiazolide carboxyl is stabilised by multiple polar interactions with Thr 313, Asn 343, and multiple water molecules. Active site Ser 61 and Ala 315 form hydrogen bonds with the *β*-lactam carboxyl. Finally, the amide oxygen interacts with Asn 149 and the thiazolide ring nitrogen with a water molecule. In our Cloxacillin complex (Fig. 3c, d), a few parallels can be drawn; for example, the thiazolide carboxyl forms H-bonds with side chain oxygens of Thr 235 and Ser 237. The amide branch of the *β*-lactam ring forms multiple H-bonds with Asn 104, Asn 132, and Ser 237. Additionally, the ligand is also stabilised by H-bonds between Asn 170 and the carboxyl oxygen of the *β*-lactam and that of Ser 130 with the nitrogen of the thiazolide ring. Within PBP-5, most of the electron density corresponding to the thiazolidine ring and carboxylate of Cloxacillin is clearly resolved, except for one of the two methyl groups at position 2 of the ring^35^. The presence of weak electron density around the variable moiety, especially for the 2-chlorophenyl ring, has previously been reported^34,35^. In contrast, we observe clear and strong density peaks surrounding the whole ligand except the 2-chlorophenyl ring (Fig. 3a, b). This might be a result of rotation on the 2-chlorophenyl ring about the C-C bond connecting it to the oxazole. To justify strong positive density peaks around the oxazole, an additional alternate conformation of the variable moiety was modelled. As a result of occupancy refinement, the major conformer has 75% and the minor conformer has 25% occupancy. Only the major conformer is referred to in the analysis and Fig. 3. Superposing our Cloxacillin complex with E. coli PBP-5 (3MZD) and the Class C *β*-lactamase AmpC (1FCM) (Fig. 6) highlights the marked differences in the ligand conformations with respect to the enzymes.

### Three distinct Dicloxacillin conformations in CTX-M-14

**Fig. 7:**
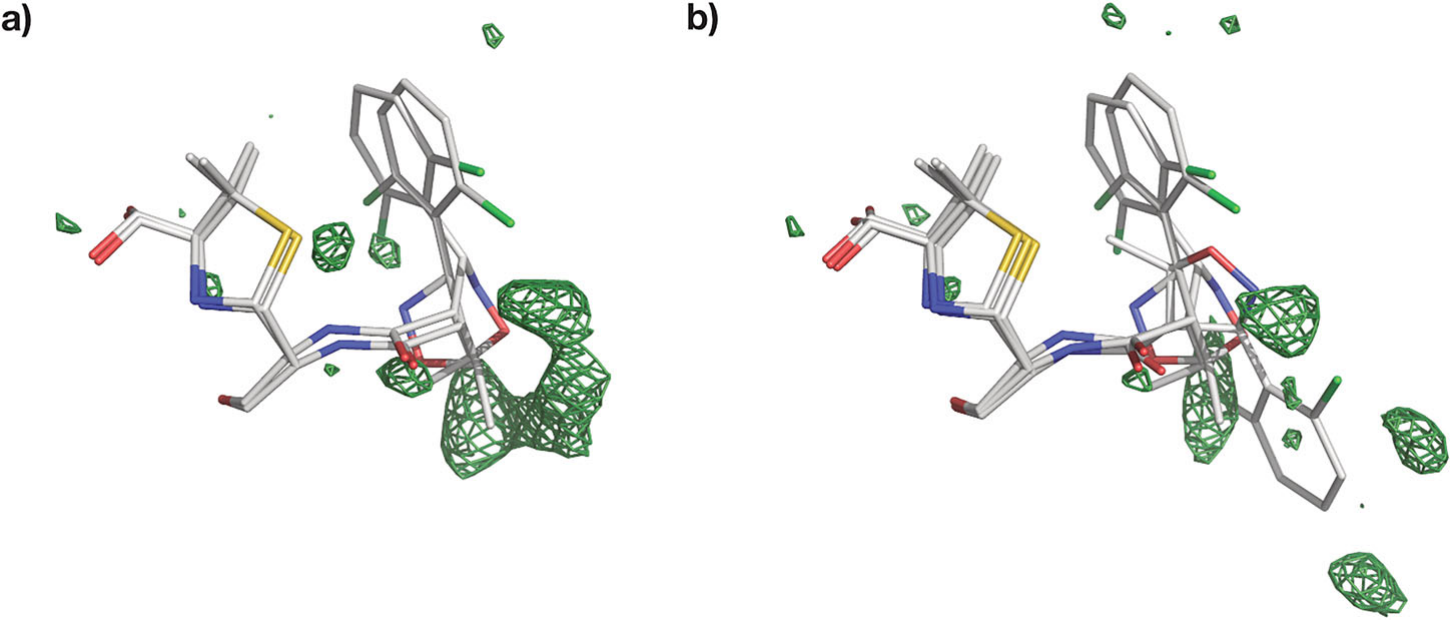
Multi-conformer structure of Dicloxacillin bound to CTX-M-14 E166A. **a** One alternate conformation along with F_*o*_-F_*c*_ map around 3 Å of the ligand at an RMSD of +3.0 (**b**) A third conformation reduces some of the persistent difference density from (**a**); F_*o*_-F_*c*_ map around 3 Å of the ligand at an RMSD of +3.0.

To the best of our knowledge, our CTX-M-14 E166A Dicloxacillin complex is the first observed 3D structure of the antibiotic with a *β*-lactamase enzyme. Dicloxacillin displays an unambiguous difference in electron density (Fig. 4a, b) and clearly binds to CTX-M-14 E166A. A careful observation of the difference map indicates the possibility of at least two more alternate conformations as a result of rotation on the 2-chlorophenyl ring about the C-C bond connecting it to the oxazole. Thus, two orientations are similar to Cloxacillin with 40% and 21% occupancies. Adding a third ring orientation (Fig. 7a, b) reduces some of the strong positive difference electron density around the ligand. However, this conformer, with an occupancy of 39%, differs from Cloxacillin and resembles Oxacillin instead (Fig. 7a, b). Such a multi-conformer Dicloxacillin model is strongly supported by the F_*o*_-F_*c*_ map as well as the POLDER omit map. Nevertheless, only a single ligand orientation (40% occupancy) is represented in Fig. 4. This Dicloxacillin conformer also interacts with the binding pocket via residues mentioned above (Fig. 4c, d). The alternate, almost equally occupied (39%) Oxacillin resembling conformer, leads to additional ligand-residue interactions, notably, the 2-chlorine interacts with the backbone oxygen of Asn 170 (2.6 Å) and backbone nitrogen of Asp 239 (2.8 Å). Similarly, the 6-chlorine interacts with the sidechain nitrogen of Asn 104 (2.6 Å). On the same note, the conformer with the least occupancy leads to additional interactions as well. The oxygen and nitrogen of the isoxazole form H-bonds with the main chain oxygen of Ser 237 (2.7 Å). Finally, the 2-chlorine and 6-chlorine interact with the side chain oxygen of Ser 237 (2.9 Å) and nitrogen of Asn 104 (2.6 Å), respectively.

### Isoxazolyl-Penicillin binding disrupts the H-bond between the *α*/*β*-domain and the *Ω*-loop

**Fig. 8:**
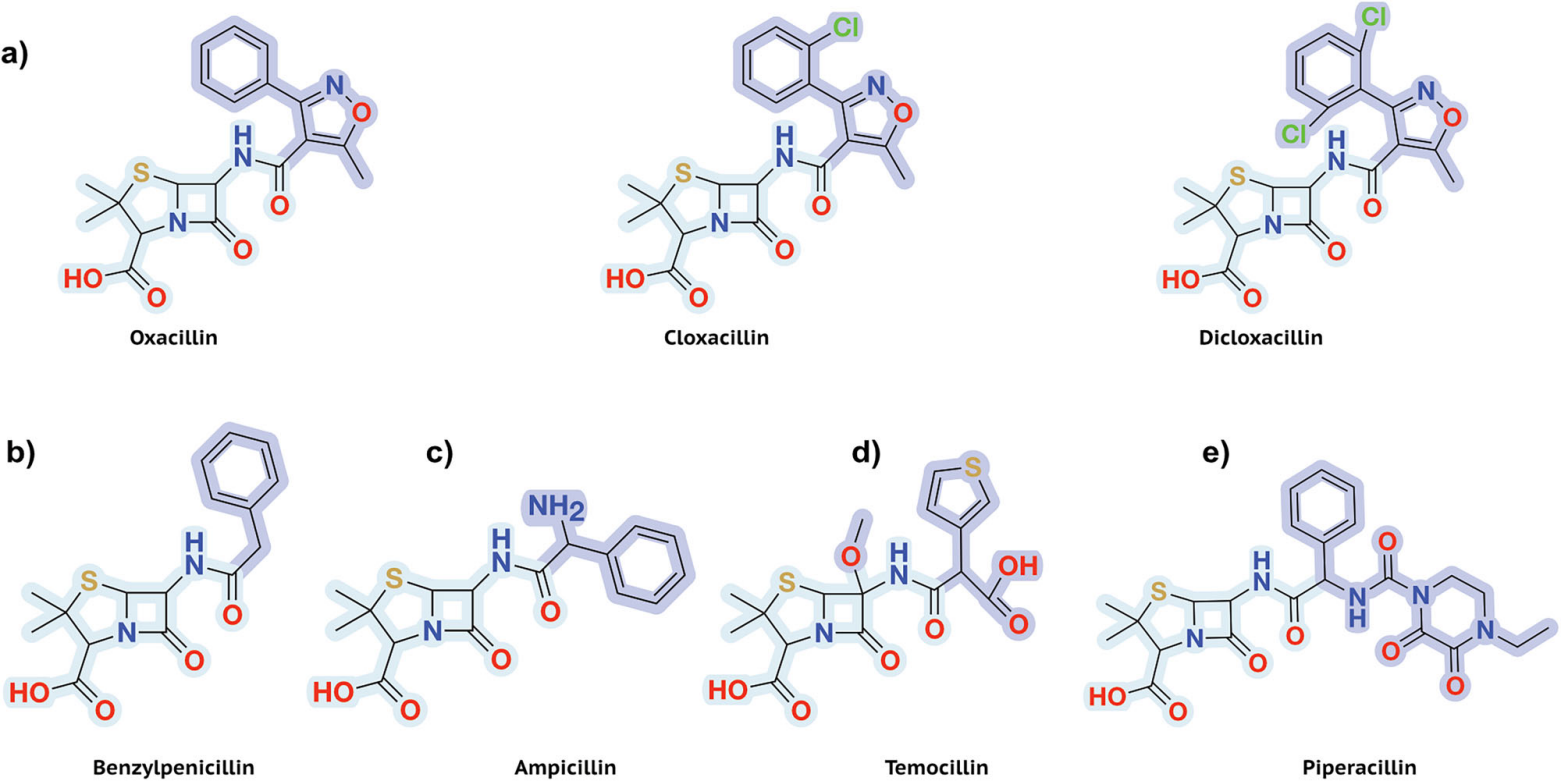
Structural comparison of various penicillins. **a** Isoxazolyl Penicillins; Oxacillin, Cloxacillin and Dicloxacillin from left to right (**b**) Natural Penicillin; Penicillin G (Benzylpenicillin) (**c**) Aminopenicillin; Ampicillin (**d**) Carboxypenicillin; Temocillin (**e**) Ureidopenicillin; Piperacillin. Variable sidechains at position C6 are highlighted.

To obtain a clear picture of the binding mode of different penicillins with class A *β*-lactamases, we compared the CTX-M-14-Isoxazolyl Penicillin complexes (Fig. 8a) with, Benzylpenicillin (Fig. 8b) in complex with CTX-M-9 S70G (3HVF)^36^; with Ampicillin (Fig. 8c) in complex with CTX-M-14 E166A (8B2O)^37^ and CTX-M-14 E166A/K234R (7K2Y)^38^; with Temocillin (Fig. 8d) in complex with CTX-M-14 wildtype (6UNB)^39^; and with Piperacillin (Fig. 8e) in complex with CTX-M-9 S70G (3Q1F)^40^. While these substrates have a common core of a *β*-lactam fused to a thiazolidine ring, they differ in the attached sidechain at position 6 (highlighted in Fig. 8). Most ligands form hydrogen bonds with conserved active site residues (70, 104, 130, 132, 170, 235, and 237). However, due to the variability at position 6, the ligand orientation as well as the interacting active site residues change with every ligand.

In the case of Piperacillin and Benzylpenicillin, both hydrolysed and unhydrolysed substrates in complex with CTX-M-9 S70G have been studied. The unhydrolysed substrates form a hydrogen bond with Lys 234, which is replaced with a water-mediated interaction between the carboxylate of the thiazolidine ring and residues Lys 234 and Arg 276, upon hydrolysis. This water-mediated contact is also observed for acyl-enzyme intermediate complexes of Ampicillin, Temocillin, and the Isoxazolyl Penicillins with CTX-M-14 presented in this study. One noteworthy difference between the Temocillin and other complexes is the orientation of Tyr 105. In the CTX-M-14 Temocillin complex, the *α*-methoxy group is stabilised by non-polar interaction with the aromatic Tyr 105^39^. This likely causes a shift of the residue towards the methoxy group, thus exhibiting an altered ring orientation compared to other CTX-M-Penicillin complexes. The active site residue Ser 237 is conserved throughout the CTX-M family and is important for substrate binding^41^. Various Penicillins show multiple sidechain conformations of this residue; in each structure, there always exists at least one conformation in which the side-chain oxygen of Ser 237 interacts with the carboxyl of the thiazolide ring.

The hydrogen bond between residues 170 and 240 connects the *Ω*-loop and the *α*/*β*-loop domain, and is conserved in almost all class A *β*-lactamases^36^. Complex formation with bulky substrates like cephalosporins requires the active site to open up and the hydrogen bond between residues 170 and 240 to rupture^36^. Interestingly, while all penicillin complexes mentioned above retain this weak hydrogen bond (3.1–3.2 Å), it is lost (3.4–3.6 Å) in the Isoxazolyl-Penicillin complexes. Consequently, the distance between C_*α*_ atoms of residues Asn 170 and Asp 240 increases by almost 0.5 Å in the case of Isoxazolyl-Penicillins in comparison to other penicillins. This is surprising considering that Piperacillin has a larger molecular volume (≈439 Å^3^) than Dicloxacillin (≈363 Å^3^)^42^. This highlights that the observed active site plasticity may not only correlate with ligand size, but also with its conformation and the nature of its sidechains.

### The conformational variability of Isoxazolyl-Penicillins might promote active site plasticity in CTX-M-14

**Fig. 9:**
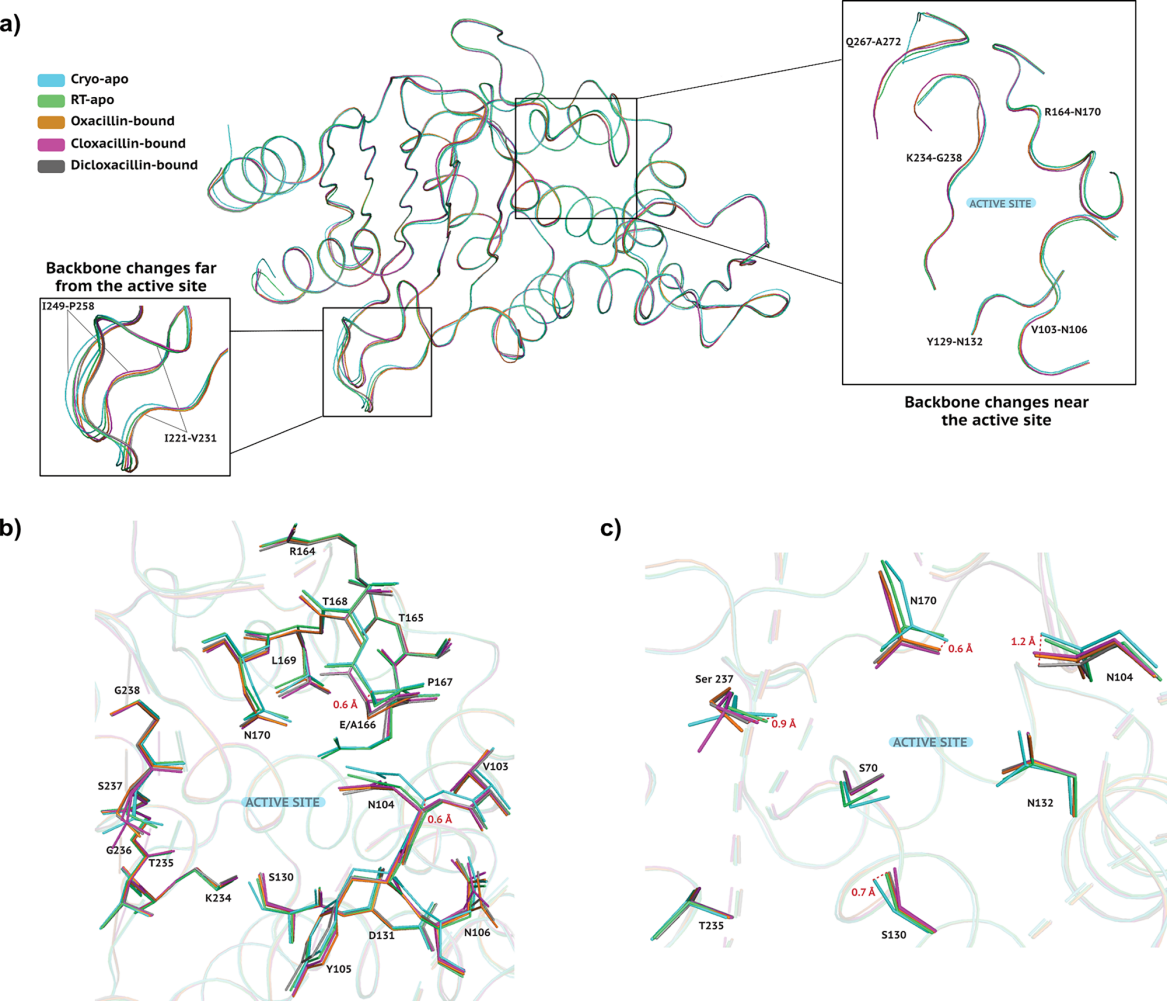
Structural comparison of apo and Isoxazolyl Penicillin-bound CTX-M-14. **a** Superposition of cryo-apo CTX-M-14 WT (cyan:6D7H), RT-SSX CTX-M-14 WT (green:7ZPV), and Isoxazolyl Penicillins bound to CTX-M-14 E166A (Oxacillin-bound: orange, Cloxacillin-bound: magenta, Dicloxacillin-bound: grey). Enlarged panels show visible backbone changes between structures around the active site (right) and away from the active site (left). **b** Examples of E166A mutation-induced backbone shift (P167) and temperature/ligand-induced backbone shift (N104) around the active site. **c** Temperature/ligand-induced differences in the sidechain conformations of active-site residues.

To verify whether the observed conformational flexibility of the chlorinated derivatives of Isoxazolyl Penicillins is correlated to the E166A mutation, we compared the respective structures to wild-type CTX-M-14 (Fig. 9a).

To avoid misinterpretation of any structural differences, if observed, we selected a cryo structure (6D7H)^43^ and a room-temperature (RT) structure (7ZPV)^44^ belonging to the same space-group as our complexes (P3_2_21). The binding pocket of class A SBLs is composed of three loops, which are part of the *α*-helical domain (residues 103–109 and 129–132), the *α*/*β*-domain (residues 234–242 and 267–272) and the *Ω*-loop domain (residues 163-176), respectively^45^. The cryo- and RT wild-type structures show subtle structural deviations in these regions (Fig. 9a: right panel). These structural differences highlight the fact that RT structures may convey a more realistic picture of proteins during and after ligand binding. For instance, loop Val 103 - Asn 106 shows slight, but noticeable displacement in the apo- as well as in the ligand-bound RT structures compared to the cryo structure (Fig. 9b). Moreover, comparing the apo-structure with all ligand-bound structures, there is an  ≈ 0.6–1.2 Å difference in the side chain nitrogen of active-site Asn 104 as well as Ser 237 Å (Fig. 9c). Interestingly, the RT apo Ser 130 aligns quite well with ligand-bound structures, unlike the cryo apo-structure (Fig. 9c).

However, not only temperature and ligand binding but also the E166A mutation seems to induce a slight backbone shift in residues Thr 165 - Leu 169 (Fig. 9b), especially in Pro 167 (0.4-0.7 Å). Nevertheless, this shift towards the phenyl ring of Isoxazolyl-Penicillins does not prevent the bulky hydrophobic moiety of the Isoxazolyl-Penicillins from being placed in its current position.

Regardless of the temperature, as mentioned above, we observe a hydrogen bond between Asn 170 and Asp 240 in all apo structures ( ≈ 3.1 Å). The loss of this hydrogen bond has previously been associated with an active site expansion to accommodate bulky substrates^36^. All three Isoxazolyl-Penicillin complexes clearly indicate a loss of this hydrogen bond. As an indirect consequence of this loss, the Asn 170 sidechain shifts by  ≈ 0.6 Å (Fig. 9c). In summary, rather than being induced by the active site mutation (E166A), the observed conformational variability of CTX-M-14 seems to be a response to the Isoxazolyl-Penicillin binding. Since a similar effect was not observed with other penicillins, we speculate that the intrinsic conformational heterogeneity of the Isoxazolyl-Penicillins promotes this behaviour.

### Chlorination increases flexibility of Isoxazolyl-Penicillin binding to CTX-M-14

**Fig. 10:**
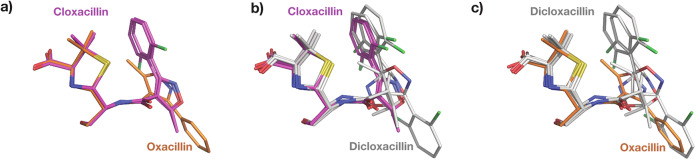
Superposed Isoxazolyls bound to CTX-M-14 E166A. **a** Oxacillin (Orange) superposed with Cloxacillin (Magenta) (**b**) Cloxacillin (Magenta) superposed with Dicloxacillin (Grey) (**c**) Dicloxacillin (Grey) superposed with Oxacillin (Orange).

We have obtained reliable structures of Oxacillin, Cloxacillin and Dicloxacillin bound to CTX-M-14 E166A mutant at room temperature, displaying strong electron density around the covalently bound Isoxazolyl Penicillins. Interestingly, at 20 °C, the electron density around Oxacillin clearly indicates the presence of a single orientation and leaves no room even for a very low occupancy alternate conformer. On the other hand, Cloxacillin and Dicloxacillin clearly adopt multiple conformations as mentioned in previous sections. The 2-chlorophenyl ring of Cloxacillin assumes one additional orientation, whereas the 2,6-dichlorophenyl ring of Dicloxacillin shows at least two alternate orientations. There is still observable minor positive and negative electron density surrounding Dicloxacillin, signatures of its partially dynamic binding mode. To avoid overfitting, we have left these minor populated states unmodelled. Unlike Oxacillin, Cloxacillin and Dicloxacillin contain chlorobenzenes, which seem to lead to an elevated dynamicity of the latter two ligands at room temperature. This could also be an example of gradual and subtle changes in ligand binding between the parent compound (Oxacillin) and its derivatives (Cloxacillin, Dicloxacillin). Interestingly, Dicloxacillin appears to adopt conformations that resemble both Cloxacillin and Oxacillin (Fig. 10). The extent to which this diversity in conformation between isoxazolyl penicillins is attributable solely to phenyl chlorination and the extent to which it is caused by temperature will be systematically investigated in further studies.

### Outlook

With the advent of methodologies like serial synchrotron crystallography (SSX), data collection close to or at physiological temperatures has become more accessible to a larger user community, as it enables effectively mitigating radiation damage^46–48^. Consequently, protein-ligand interaction studies can become more insightful. Room temperature data collection removes vitrification artefacts and ice formation, enabling observations of conformational heterogeneity. Despite a somewhat lower throughput than other sample delivery methods, fixed-target SSX is very material efficient and reduces radiation damage effects by distributing the dose over thousands of crystals^46–50^. Additionally, our environmental control system provides constant high relative humidity and prevents non-isomorphism caused by dehydration^24^. Collectively, these aspects mitigate cryo-crystallography artefacts, and allow to study of enzyme-ligand interactions, e.g., for structure-based drug design. Nevertheless, SSX’s potential in the realm of drug discovery has not yet been extensively explored.

Recent studies have utilised serial crystallography for fragment screening and ligand binding campaigns, successfully reporting additional binding sites and allosteric conformational responses^51–53^. Similarly, we have set ourselves the goal of elucidating the binding mode of existing drugs at physiologically relevant temperatures. Our structures not only provide insights into the binding of an Isoxazolyl-Penicillin, but also enable a systematic comparison of the binding mode of the various derivatives. This direct comparison highlights that the conformational flexibility correlates not only with the chemical modification but also with the plasticity of the active site of CTX-M-14. The binding of Isoxazolyl- Penicillins to CTX-M-14 disrupts a key hydrogen bond between the *Ω* loop and the *α*/*β*-domain, a change that coincides with active-site expansion needed to accommodate bulky methoxyimino-cephalosporins as reported previously. This finding could open up new avenues for the development of new inhibitors or antibiotics, particularly for the most widespread *β*-lactamase CTX-M-14 or other ESBLs.

These examples highlight the advantages of room-temperature crystallography for protein-ligand analyses and studies designed to support structure-based drug design. We hope that our room-temperature study can serve as a template for further protein-ligand interaction analyses and that a better understanding of the conformational plasticity of both the substrate and the ligand will support future drug-design campaigns.

## Methods

### Protein purification and sample preparation

The CTX-M-14 E166A gene was synthesised and cloned into a pET-24a(+) vector (BioCat GmbH, Heidelberg, Germany) with a Kanamycin selection marker. The plasmid was transformed into E. coli strain BL21 (DE3) and grown in LB (Luria Miller) medium supplemented with 50 *μ* g/mL Kanamycin at 37 °C until an OD_600_ of 0.6–0.8 was reached. Protein expression was induced by the addition of IPTG to a final concentration of 175 *μ* M, after which the cells were incubated at 37 °C for 4 h. The cells were harvested by centrifugation (5500 × g, 10 min, 4 °C) and pellets were stored at −20 °C until purification. For purification, the cell pellet was resuspended in purification buffer (20 mM MES, pH 6), followed by sonication for cell lysis. Cell debris was separated by centrifugation (20,000 × g, 1 h, 4 °C). The cleared supernatant was dialysed overnight against a large volume of purification buffer at 4 °C using a 6-8 kDa molecular weight cut-off membrane. The CTX-M-14 protein was purified using cation exchange chromatography (5 ml HiTrap SP FF, Cytiva) and eluted using a gradient of 20 mM MES, pH 6, 0−50 mM NaCl over 5 column volumes. The protein was then concentrated to 22 mg/ml using 10 kDa centrifugal filter units (Amicon Ultra-15). For crystallisation, the CTX-M-14 E166A solution (22 mg/ml) was mixed with 45% (v/v) crystallising agent (40% (w/v) PEG 8000, 200 mM LiSO_4_, 100 mM sodium acetate, pH 4.5) and with 5% (v/v) undiluted seed stock solution to induce micro-crystallisation. This resulted in crystals with a homogeneous size distribution of ca. 11−15 *μ* m overnight. For soaking experiments, Oxacillin, Cloxacillin, and Dicloxacillin were dissolved in the stabilisation buffer (28% (w/v) PEG 8000, 140 mM LiSO_4_, 70 mM sodium acetate, 6 mM MES pH 4.5) to reach a solubility of about 500 mM each. The ligands did not dissolve completely; hence, the supernatant was mixed with the crystal slurry in a 3:1 ratio. Our efforts to soak Flucloxacillin into CTX-M-14 E166A crystals, unfortunately, remained unsuccessful.

### Ambient temperature data collection

Serial diffraction data were collected at the EMBL endstation P14.2 (T-REXX) at the PETRA-III synchrotron (DESY, Hamburg, Germany) with an X-ray beam of 10 × 7 *μ*m (H × V) on an Eiger 4M detector (Dectris, Baden-Daettwil, Switzerland). Before sample loading, the HARE-chip was glow-discharged to increase surface hydrophilicity and thus, improve sample-loading homogeneity^54^. Roughly 120 *μ*l ligand-soaked microcrystal slurry (90 *μ*l ligand solution mixed with 30 *μ*l crystal slurry) was then pipetted onto the fixed target HARE-chip containing 20,736 wells, in a humidified (98%) environment. Data collection was conducted as previously described^55^ within the environmental control box^24^ mounted on the T-REXX endstation. Briefly: After mounting, the chips were raster-scanned through the X-ray beam using a 3-axis piezo translation stage setup (SmarAct, Oldenburg, Germany)^54^. Data collection was carried out at 20 °C with the relative humidity controlled at 98%. For reasonable data statistics, approximately 5000 to 11,000 still diffraction images were recorded per structure, as previously determined as a broad guideline^46,56^.

### Data-processing and structure refinement

Diffraction data were processed using the CrystFEL v0.09.1 package^57^. Structures were solved by molecular replacement using PHASER^58^ with our previously determined CTX-M-14 structures as a search model with one molecule in the asymmetric unit (PDB-ID: 6GTH). Structures were refined with iterative cycles of *phenix.refine* and manual model building in COOT-v0.9^59–61^. Figures were generated with PyMOL^62^ and ChemDraw (Revvity Signals Software, Inc., Waltham, USA). Data collection and refinement statistics are summarised in Table 1.

### Data analysis

To illustrate localised differences in ligand binding, the structures were superimposed using the PyMOL function "super mobile, target, [object = name]", which does sequence-dependent alignment and iterative RMS refinement, typically always aligning conserved secondary structure. Since all *β*-lactamases across classes A, C, and D have well-conserved secondary motifs^45^, this allowed a systematic comparison of the ligands in their respective binding pockets irrespective of the enzyme classes.

### Reporting summary

Further information on research design is available in the Nature Portfolio Reporting Summary linked to this article.

## Supplementary information

Reporting Summary

## Data Availability

The structural data obtained for the present study are available in the Protein Data Bank archive (https://www.rcsb.org/) under accession codes: 9R0R, 9RAO, 9RAJ. Further details are available in Table 1.
